# Lack of association between Chlamydia Pneumoniae serology and endothelial dysfunction of coronary arteries

**DOI:** 10.1186/1476-7120-3-12

**Published:** 2005-04-27

**Authors:** Markus Ferrari, Gerald S Werner, Barbara M Richartz, Albrecht Oehme, Eberhard Straube, Hans R Figulla

**Affiliations:** 1 Clinic of Internal Medicine I, Friedrich-Schiller-University, D – 07740 Jena, Germany; 2 Institute of Microbiology, Friedrich-Schiller-University, D – 07740 Jena, Germany

## Abstract

**Background:**

Recent publications brought up the hypothesis that an infection with Chlamydia Pneumoniae (CP) might be a major cause of coronary artery disease (CAD). Therefore, we investigated whether endothelial dysfunction (ED) as a precursor of atherosclerosis might be detectable in patients with previous infection with CP but without angiographic evidence of CAD.

**Methods:**

We included 16 patients (6 male / 10 female) of 52 consecutive patients with normal coronary angiography who had typical angina pectoris and pathologic findings in the stress test. Exclusion criteria were: active smoker, elevated cholesterol, hypertension, age > 65 years, diabetes mellitus, treatment with ACE-inhibitors, or known CAD. Blood sample analysis for serum titer against CP (aCP-IgG) was performed after coronary angiography. We looked for endothelial dysfunction analyzing the diameter of the left anterior descending coronary artery (LAD) before and after acetylcholine (ACh) i. c. Quantitative analysis of luminal diameter (LD) was performed in at least two planes during baseline conditions and after ACh for 2 minutes in dosages of 7.2 μg/min and 36 μg/min with an infusion speed of 2 ml/min. Using Doppler guide wire, the coronary flow velocity was measured continuously in the LAD. The coronary flow velocity reserve (CFVR) was measured after 20 μg adenosine i. c.

**Results:**

10 patients had an elevated aCP-IgG (> 1:8). 6 patients with negative titers (aCP-IgG ≤ 1:8) served as control (CTRL). Both groups were comparable in age, gender, angina class, results of non-invasive stress-test and the baseline values of LD and flow. In the CP positive group 3 patients (30%) did not show an increase of LD after ACh as evidence of ED. In the CTRL group 4 patients (67 %) had ED. There was no association between aCP-IgG and changes of coronary blood flow after ACh. All patients showed normal CFVR (3.0 ± 0.27) irrespective of their aCP-IgG values.

**Conclusion:**

In patients with typical symptoms of coronary ischemia but without angiographically visible CAD and absence of other factors affecting the endothelial function, a previous infection with CP is not associated with endothelial dysfunction.

## Introduction

If coronary angiography is carried out due to pathological stress-test or angina pectoris, 10% to 20% of the patients do not reveal any atherosclerotic alteration of the coronary vessel related to the clinical symptoms [1]. As a possible explanation of this phenomenon, an infectious mechanism was discussed, which leads to an endothelial dysfunction (ED) and thus functional impairment of the coronary circulation [2,3].

The intracellular bacterial pathogen Chlamydia pneumoniae (CP) causes respiratory tract infections of increasing incidence with age [4]. The proof of CP both in atherosclerotic coronary vessels at post-mortem examinations and also in tissue samples from coronary atherectomy brought up the hypothesis that an infection with CP is also an important promoter of atherosclerosis and CAD [5-7]. In addition, the successful treatment with antibiotics of patients suffering from unstable angina pectoris supported this hypothesis [8,9]. CP was accused of damaging the coronary endothelial cells and therefore causing a local inflammatory reaction and promoting the sub-endothelial storage of low density lipoprotein (LDL) cholesterol [10-12].

The ED can be regarded as an early form of CAD before the detection of angiographically visible alterations caused by storage of cholesterol in the vessel wall [13]. The lack of dilatation of the coronary vessels during infusion of acetylcholine (ACh) uncovers an ED in vivo. This method of inducing a paradoxical reaction of the artery was well evaluated in patients suffering from diabetes mellitus, hypercholesterolemia, obesity, hypertension, or CAD, and in smokers [14-17]. Under the medication of ACE- inhibitors an ED can be attenuated [18].

Therefore, we designed this study including only patients without any known factor which could influence the endothelial function except previous CP infection indicated by elevated antibodies. It was our aim to prove whether there is an association between an infection with CP and an ED in those patients who did not carry any of the known risk factors. A positive result would corroborate the hypothesis of a causal role of CP in atherogenesis.

## Methods

### Patient selection

All patients had to give written informed consent. The study was performed with approval of the local ethical committee of our university. Out of 1144 consecutive patients who were brought to an elective coronary angiography because of typical angina pectoris or a pathological stress test for the first time, 52 caucasian patients who fulfilled the following criteria were screened for this study during a period of 22 months. Exclusion criteria were myocardial infarction, unstable angina, ECG abnormalities at rest, disorders of wall motion or thickened left ventricular wall in echocardiography, vitiae of the valves, age ≥ 65 years, arterial hypertension (systolic blood pressure at rest ≥ 140 mmHg), any type of diabetes mellitus, obesity (body mass index ≥ 30), hypercholesterinemia (total cholesterol ≥ 5.0 mmol/l or LDL cholesterol ≥ 3.0 mmol/l), hypetriglyceridemia (triglycerides ≥ 5.0 mmol/l), and a history of smoking during the last 10 years. An acute infection represented by fever, elevated C-reactive protein (CRP > 5 mg/dl), or elevated white blood cell count (≥ 10,000 / μl) caused exclusion from the study before coronary angiography. We also excluded all patients who were on medication with ACE inhibitors, anti-hypertensive drugs, nitrates, hormones, or lipid lowering agents. The patients gave their written consent for the study 24 hours before they were transferred to the catheter laboratory.

### Study protocol

Coronary angiography was performed in standard fashion via femoral approach with a 5F diagnostic catheter. We saw atherosclerotic lesions of at least one coronary artery in 34 patients (65.4%). They were excluded and an angioplasty was carried out where necessary. The following measurements were performed in the remaining 18 patients (34.6%) in whom we did not find any alterations of the coronary arteries (exclusion of atherosclerotic plaque in all projections).

After intra coronary administration of 10.000 IE heparin, we placed a 0.014 inches Doppler guide-wire (Flowire™, Cardiometrics, USA) in the mid LAD. The tip of the wire was positioned in a straight vessel segment at least 1 cm from a departure of any relevant side branch. In 2 patients we stopped the testing due to complications: one patient showed a coronary spasm after insertion of the Doppler wire, another patient developed a bradycardia of 35 beats / min and a pressure drop immediately after starting the ACh infusion. After removal of the wire and termination of the ACh infusion, these complications were completely reversible within a few seconds. However, these two patients were excluded from further examinations and from the study. In the remaining 16 patients (30.8 %) the measurements according to the study protocol were carried out without any complications.

The luminal diameter (LD) was measured 5 mm distal to the tip of the Doppler wire in two orthogonal planes by means of automatic quantitative coronary angiography (QCA). Automatic contour detection was performed with a geometric edge differentiation technique [19]. For prevention of a study bias, the automated QCA was performed by an independent cardiologist without knowledge of the antibody titer against Chlamydia pneumoniae. Each coronary angiography was performed in the same projection as the baseline procedure.

After recording a stable Doppler baseline signal, we infused acetylcholine (ACh) (Miochol™; CIBA Vision, Switzerland) at a dose of 7.2 μg/min through the catheter into the LAD for two minutes. Assuming a blood flow in the LAD of 80 ml/min, we estimated a final ACh concentration in the coronary bed of 1/2 × 10^-6 ^mol/l (= dose 1, ACh D1). Careful attention was paid to the calculation of catheter dead space to ensure accurate delivery of acetylcholine to the coronary ostium. Subsequently, coronary angiography was carried out in the same projection as at the baseline. The ACh infusion was stopped for at least 3 minutes before proceeding. After reaching stable baseline conditions, i. c. infusion of 2 ml/min of ACh in a concentration of 36 μg/min aiming at a concentration of 1/4 × 10^-5 ^mol/l (= dose 2, ACh D2) was started for another 2 minutes. Angiography was repeated immediately after each infusion.

After another recovery period of 3 minutes, we gave a continuous infusion of saline (NaCl 0.9 %) with an infusion rate of 2 ml/min. After restoring the baseline following the angiography, we injected 0.2 mg of nitrotriglycerin (NTG) i. c. and performed a final coronary angiography in the same projections as before.

The average coronary peak flow velocity (APV) was registered continuously by means of a Doppler wire during the complete examination. Attention was paid to insure that APV had returned to baseline values between each measurement. At the end of the examination we injected 20 μg adenosine i.c. recording the maximum rise of APV in comparison to the baseline value which yields the coronary flow velocity reserve (CFVR). The CFVR measurement was carried out at least twice, and the mean value was calculated for further analysis.

### Calculation of coronary blood flow (CBF)

Coronary blood flow (CBF) was calculated out of the average peak flow velocity (APV) measured by Doppler guide-wire in the mid LAD. By offline analysis with QCA we calculated the average LD distal to the tip of the Doppler wire. Multiplying the square of 0.5 × LD with π we obtained the luminal area (LA) of the vessel at the site of measurement. LA × APV × 0.5 results in the CBF. This method of calculation of CBF has been evaluated in vitro and in vivo [20,21].

### Serological analysis of antigenic titers against Chlamydia pneumoniae

Following the catheter examination we took 10 ml of blood for the antibody determination against CP. Testing for anti-CP antibody (IgG) we used a standard micro-immuno-fluorescence (MIF) test on consecutive dilution rows. We used the MIF serology with the antigens Chlamydia pneumoniae (TW183) antigen and Chlamydia trachomatis (type D) in order to exclude cross reacting antibodies. The serum tests were performed in a double-blinded setting. The researcher who performed the serological analysis did not receive any knowledge of the results of the coronary angiography and the flow measurements and vice versa. The patients were retrospectively allotted in two groups according to their aCP-IgG values: Those patients with an aCP-IgG ≤ 1:8 formed the group of CP negative patients, individuals with aCP-IgG > 1:8 were included in the CP positive group since they were supposed to suffer from a previous infection with CP. The results of the Chlamydia trachomatis MIF test were considered in this grouping.

### Statistical analysis

All values are expressed as mean ± standard deviation (SD). P values were calculated using a two-tailed student's t test for statistical analysis of continuous variables by Excel™ (Mircosoft Co., USA). Multivariate logistic regression analysis including aCP-IgG, sex and age as risk factors of ED and two-tailed exact Fisher's test were performed using SPSS™ (SPSS Inc., USA). A p value ≤ 0.05 was considered statistically significant.

## Results

The analysis of the serum probes showed increased aCP-IgG values (>1:8) in 10 patients (62.5%). They formed the subgroup of the CP positive individuals. The remaining 6 patients (37.5%) had negative aCP-IgG titers (≤ 1:8) and served as the control group. All 16 patients in the study were negative (≤ 1:8) against Chlamydia trachomatis (type D) in the MIF test.

The baseline data of all patients are summarized in table 1. The results of the testing in the catheter laboratory are listed in table 3. Coronary blood flow velocity was 18.9 ± 4.49 cm/sec in our study population. The APV increased to 35.0 ± 12.08 cm/sec during middle dosage and 42.1 ± 11.49 cm/sec during the infusion of the high dosage of ACh. After the bolus injection of 0.2 mg NTG, we measured an APV of 27.7 ± 8.22 cm/sec. There was no significant difference in the APV values between the 2 groups.

**Table 1:** Baseline characteristics of study patients, and number of patients with pathologic findings in bicycle or scintigraphy stress-test.

	**All patients**	CP positive	**CP negative**
**Number**	16	10 (62.5 %)	6 (37.5 %)
**Age **(years)	50.7 ± 7.02	53.2 ± 6.48	47.0 ± 6.08
**Sex **(male)	6 male (37.5 %)	4 male (40 %)	2 male (33.3 %)
**Angina class **(CCS)	2.4 ± 0.48	2.4 ± 0.49	2.3 ± 0.47
**Pathologic ECG in bicycle stress-test**	12 (75 %)	7 (70 %)	5 (83.3 %)
**Pathologic scintigraphy**	5 (31.3 %)	3 (30 %)	2 (33.3 %)
**Total Cholesterol levels**	4.0 ± 0.58 mmol/l 154.1 ± 22.41 mg%	4.1 ± 0.68 mmol/l 157.5 ± 26.48 mg%	3.8 ± 0.28 mmol/l 148.4 ± 10.87 mg%
**Systolic blood pressure**	121 ± 12.0 mmHg	120 ± 11.7 mmHg	122 ± 12.4 mmHg
**Smoking history (last 10 years)**	All negative	All negative	All negative

Total Cholesterol levels were below 200 mg% (5.2 mmol/l) in all patients, the systolic blood pressure did not exceed 139 mmHg in any case.

CP: Chlamydia pneumoniae {values are mean ± SD, % of each group}.

**Table 3:** Coronary blood flow and vessel diameter.

	**All patients**	CP positive	**p**	**CP negative**
**CBF **(ml / min) ^(+/- percentchange)^				
**baseline**	22.6 ± 14.39	22.0 ± 13.80	→ 0.503 ←	23.4 ± 15.28
**ACh D1**	39.6 ± 21.21 ^(+75.2%)^	40.4 ± 22.34 ^(+83.6%)^	→ 0.172 ←	38.5 ± 19.33 ^(+64.5%)^
**ACh D2**	48.7 ± 30.34 ^(+115.5%)^	51.6 ± 27.82 ^(+134.5%)^	→ 0.177 ←	41.3 ± 34.78 ^(+76.5%)^
**NaCl (control)**	23.1 ± 12.15 ^(+2.2%)^	22.1 ± 12.38 ^(+0.4%)^	→ 0.697 ←	24.7 ± 11.57 ^(+5.5%)^
**NTG**	40.3 ± 23.06 ^(+78.3%)^	39.5 ± 20.74 ^(+79.5%)^	→ 0.696 ←	41.0 ± 24.74 ^(+75.2%)^
Vessel diameter **(mm)** (+/- percent change)				
baseline	2.53 ± 0.50	2.55 ± 0.50	→ 0.773 ←	2.51 ± 0.48
ACh D1	2.55 ± 0.50 ^(+0.8%)^	2.56 ± 0.52 ^(+0.4%)^	→ 0.658 ←	2.54 ± 0.46 ^(+1.2%)^
ACh D2	2.54 ± 0.61 ^(+0.3%)^	2.60 ± 0.62 ^(+2.0%)^	→ 0.289 ←	2.46 ± 0.58 ^(-2.0%)^
NaCl (control)	2.54 ± 0.45 ^(+0.1%)^	2.52 ± 0.48 ^(-1.1%)^	→ 0.543 ←	2.58 ± 0.39 ^(+2.7%)^
NTG	2.88 ± 0.52 ^(+13.8%)^	2.88 ± 0.47 ^(+13.0%)^	→ 0.801 ←	2.89 ± 0.60 ^(+15.1%)^
**CFVR**	3.0 ± 0.27	3.1 ± 0.31	→ 0.652 ←	3.0 ± 0.10

Coronary blood flow (CBF) and vessel diameter before (baseline) and after i. c. infusion of acetylcholine (ACh) at medium dose (ACh D1: 7.2 μg/min → 1/2 × 10^-6 ^mol/l) and high dose (ACh D2: 36 μg/min → 1/4 × 10^-5 ^mol/l), 0.2 mg nitroglycerin (NTG), continuous infusion of saline (NaCl 0.9 % at 2 ml/min) served as control. The comparison of the CBF, and of the vessel diameter with the corresponding baseline value is expressed as percent change. CFVR: coronary flow velocity reserve (adenosine i. c. versus baseline), CP: Chlamydia pneumoniae. {values are mean ± SD, p values of two-tailed student's t-test}

**Figure 1:**
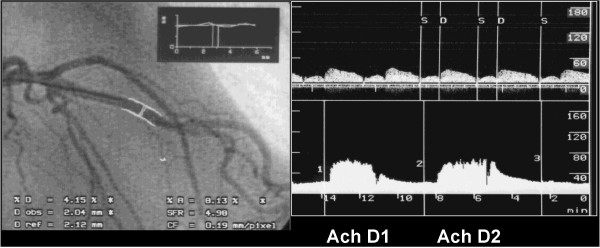
Decreased vessel diameter (62% of baseline value) during acetylcholine (36 μg/min) i. c. Continuous recording of flow velocity in this Chlamydia pneumoniae positive patient after i. c. ACh infusion (ACh D1: 7.2 μg/min, ACh D2: 36 μg/min).

**Figure 2:**
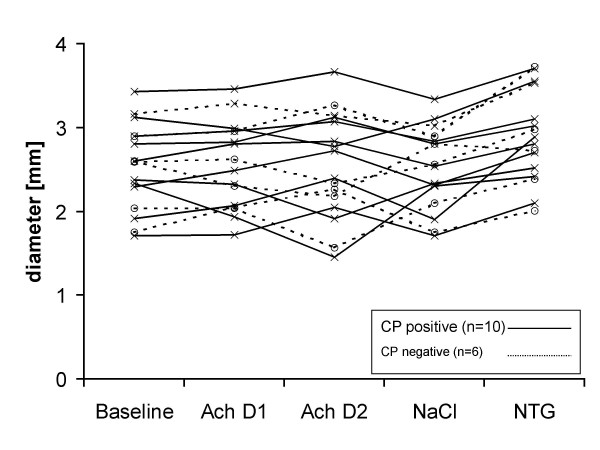
Vessel diameter of all 16 patients at baseline, during i. c. infusion of acetylcholine (ACh) with 7.2 μg/min (ACh D1), and with 36 μg/min (ACh D2), during NaCl (0.9% 2 ml/min i. c.), and after 0.2 mg nitrotriglycerin (NTG) i. c. Patients without an elevated IgG serum titer against Chlamydia pneumoniae (CP negative) are presented by dotted lines.

Among the negative control group without previous contact with CP, four patients (66.7%) did not show any increase of luminal diameter (LD) during ACh infusion, what was judged as an ED (Figure 1). Three patients (30%) of the CP positive subgroup did not show an increase of the LD (Figure 2). The two-tailed exact Fisher's test did not unveil any significant difference between the two groups (p = 0.302) as shown in table 2. For the final measurement of the CFVR, we recorded a 2.5 to 3.5 fold increase of the APV (3.0 ± 0.27) after i.c. injection of 20 μg adenosine. There was no association between the occurrence of an ED and the height of the CFVR (p = 0.258) or between the aCP-IgG and the CFVR values (p = 0.735). We did not find any correlation between CFVR and the flow velocity increase during high dose ACh infusion (r = -0.19).

## Discussion

In this prospective study we investigated whether a previous infection with Chlamydia pneumonia is associated with an endothelial dysfunction of the coronary vessels before the presence of atherosclerosis. It was our intention to exclude all patients with known risk factors for atherosclerosis and endothelial dysfunction (e.g. hypertension, hyperlipidemia, smoking, and diabetes). In addition, those patients who took medication which could have influenced the endothelial function were excluded. Sixteen patients without any angiographically visible atherosclerotic alteration but typical signs of coronary ischemia at stress were included in the study. We evaluated the capability of the coronary endothelium to promote a vessel dilatation in this highly selected group of patients. A lack of flow mediated diameter increase of ≥ 5% was interpreted as an ED. Since we did not find a higher incidence of ED in those patients who had a previous CP infection than in the patients in the CP negative control group, we disproved our hypothesis that CP induces an endothelial damage or even disturbs the endothelial function before visible atherosclerotic changes take place.

We observed a relatively large proportion of so-called "exclusion of CAD" among the 52 patients who were screened according to the strict inclusion criteria. Previous angiography studies reported a prevalence of negative coronary angiograms of 10% to 20% in comparable populations [1]. In contrast to predominantly male patient populations in catheter laboratories, we viewed two thirds of female individuals in our study collective. Women show negative coronary angiograms more often than men [22,23]. Furthermore, our patients were relatively young which may also explain this relatively large proportion of negative coronary angiograms. On the other hand, all patients suffered from typical symptoms of coronary ischemia such as angina pectoris, ECG changes, or pathologic findings in non-invasive stress test. Another limitation of our study is the relatively low sample size due to difficulties in recruiting patients. We screened 1144 patients for the study, but only 18 patients fulfilled the strict inclusion criteria.

**Figure 3:**
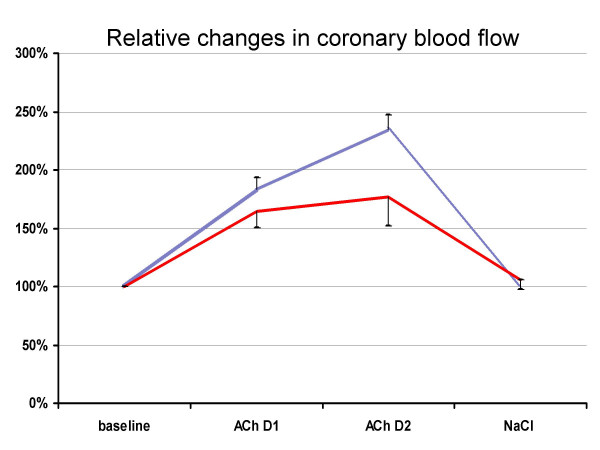
Relative changes in coronary blood flow (+ / - standard error of mean) The dotted line presents patients with previous infection with Chlamydia pneumoniae (CP). The bold line shows the results in the CP negative patients. The curves represent the relative changes compared to baseline during i. c. infusion of acetylcholine (ACh) with 7.2 μg/min (ACh D1), with 36 μg/min (ACh D2), and during NaCl (0.9% 2 ml/min i. c.)

The CP positive group and the CP negative group were comparable in terms of age, gender, angina class, and non-invasive stress-test results. The tendency of a higher proportion of women to be CP positive was statistically insignificant (p: 0.172). However, there was no significant difference between the two groups regarding vessel diameter and flow velocity at the baseline and after administration of nitroglycerin. The average baseline APV of 18.9 ± 4.49 cm/s with an increase to 27.7 ± 8.22 cm/s is consistent with values reported in the literature [24]. During ACh infusion, we even recorded a higher increase of LD in patients with elevated aCP-IgG compared to the CP negative individuals, but there was no significant difference here either (Figure 3). We observed a lack of vasodilatation during ACh in 67 % of the CP negative and 30 % of the CP positive patients (p 0.302). Therefore, we were not able to verify the hypothesis that a previous infection with CP leads to a higher incidence of endothelial dysfunction among patients with normal coronary arteries.

The increase of APV to 300 % after adenosine was recorded among all patients irrespective of the presence of ED or the CP titer status. An average CFVR of 3.0 ± 0.27 indicates normal coronary blood flow regulation of the arteriolar coronary vessels of the study patients [24]. We conclude that a previous infection with CP does not influence the dilatory capacity of the arteriolar bed of the coronary circulation represented by the CFVR.

Non of our patients showed any signs of atherosclerotic lesions of a coronary vessel in the angiogram. Intra-vascular ultrasound would have been more sensitive to uncover changes of the integrity of the vessel wall in some patients despite a negative coronary angiogram [25]. However, the endothelial function tested with ACh is one of the most sensitive methods for detecting early damage of the endo-vascular structure on the basis of physiologic function [26].

In patients suffering from CAD, the presence of ED predicts an enlarged cardiovascular event rate [27]. Since atherosclerosis is considered to be a chronic inflammation of the artery vessel wall, it should be the interaction of CP with cells of the vasculature that can result in a local inflammatory response. It was reported that CP positive patients are in danger of having a higher cardiovascular event rate [8]. Furthermore, supporting the hypothesis of amplification of the progression of atherosclerosis by misbalance in the immune response, a correlation between high levels of aCP-IgG and acute coronary syndrome was demonstrated recently in a study on 830 patients [28]. Chlamydia pneumoniae infected patients with coronary artery sclerosis showed higher re-stenosis rates after a balloon angioplasty, but not after a coronary stent implantation [29]. However, we did not uncover an increased number of patients with ED among the individuals with CP antibodies. Therefore, we postulate that CP is not envolved in the early atherosclerotic cascade. The persistent CP infection of immune cells (T-cells, monocytes and macrophages) and non-immune cells (endothelial cells and smooth muscle cells) may contribute to a cascade of inflammatory mediators leading to an enhanced tissue remodeling of the arterial intima and instability of atherosclerotic plaques [30-33]. We would therefore conclude that a chronic infection with CP is not a major cause of coronary artery disease. The patho-physiologic link between CP infection and arteriosclerosis I still under discussion [34]. Recently published trials including more than 8,000 patients did not show any evidence for the hypothesis that coronary artery disease is caused by an infection with CP and antibiotic therapy is indicated [35,36]. The present model includes the involvement of CP in the secondary phase of atherogenesis including inflammation, fibrous plaque formation, plaque rupture, and thrombosis. Recent meta-analysis questioned any relevance of a CP infection in the early course of CAD [37].

## Conclusion

To our understanding of the mechanism of CP involvement in atherogenesis, we interpret the results of our study in combination with the present knowledge about CP as favoring a possible contributory rather than a causal role. However, there is need for further studies to enlighten the early pathomechanisms of chlamydial infection of the endothelium, including signaling pathways in the genesis and the progression of coronary artery disease.

**Table 2:** Changes of vessel diameter after i. c. infusion of acetylcholine (ACh D2: 36 μg/min) compared with baseline diameter.

n = 16	CP IgG < 1:16 (CP negative)	CP IgG ≥ 1:16 (CP positive)
Increase of vessel diameter	2 (33 %)	7 (70 %)
No increase of vessel diameter	4 (67 %)	3 (30 %)

The two groups were allotted according to their Chlamydia pneumoniae (CP) IgG serum titer. Two-tailed exact Fisher's test did not show any significant difference between both groups (p = 0.302).
